# Dietary source of saturated fat and percentage body fat of patients with type 2 diabetes mellitus: A cross‐sectional study

**DOI:** 10.1002/fsn3.853

**Published:** 2018-11-15

**Authors:** Camila Kümmel Duarte, Ana Luiza Teixeira dos Santos, Claudia Kirst, Graziela da S. Nunes, Karine de Franceschi, Mirela Jobim de Azevedo, Themis Zelmanovitz

**Affiliations:** ^1^ Nutrition Departament of Escola de Enfermagem Universidade Federal de Minas Gerais Belo Horizonte Brasil; ^2^ Endocrine Unit of Hospital de Clínicas de Porto Alegre Universidade Federal do Rio Grande do Sul Porto Alegre Brazil

**Keywords:** adiposity, cooking oil, diabetes mellitus, dietary fat, percentage body fat, red meat, saturated fat

## Abstract

**Background:**

The influence of dietary fat on the body fat of patients with diabetes is not well established. This cross‐sectional study aimed to analyze the association between percentage body fat (PBF) and dietary sources of fat from the usual diet of patients with type 2 diabetes.

**Methods:**

Outpatients were submitted to PBF evaluation estimated by bioelectrical impedance. The patient's usual diet was assessed by a 3‐day weighed diet record (WDR), and compliance was analyzed by comparing the protein intake estimated from the WDR and that from 24‐hr urinary nitrogen output.

**Results:**

A total of 188 patients with type 2 diabetes (aged 62.5 ± 8.8 years; 57% female, body mass index [BMI] 29.3 ± 3.8 kg/m²) were analyzed and divided into groups with high and low PBF according to mean PBF (men: 26.6 ± 7.1%; women: 39.8 ± 5.9%). Patients with high PBF consumed an increased proportion of red meat (52.0% of total meat), processed meat (5.4%), and saturated fat from red meat (2.1% of energy) compared to low PBF individuals (42.3% [*p* = 0.036]; 3.0% [*p* = 0.010]; 1.5% of energy [*p* = 0.032], respectively). According to Poisson's regression, the consumption of red meat (PR = 1.008 [95% CI = 1.002–1.013]; *p* = 0.006) and the reuse of frying oil (PR = 1.670 [95% CI = 1.240–2.249]; *p* = 0.001) were associated with higher PBF. In the adjusted analysis, the upper tertile of processed meat intake was associated with higher PBF (PR = 1.522 [95% CI = 1.226–1.891]; *p* = 0.001) compared to the lower tertile.

**Conclusions:**

The present study suggested that a higher ingestion of dietary sources of saturated fat was associated with high PBF in patients with type 2 diabetes.

## 1 BACKGROUND

Globally, overweight and obesity represent a rapidly growing threat to health (World Health Organization, 2000). Increased adiposity is a traditional risk factor for several chronic diseases, such as type 2 diabetes and cardiovascular disease (Wilson, D'Agostino, Parise, Sullivan, & Meigs, 2005). Body mass index (BMI) is the most widely used measure to evaluate adiposity; however, it does not assess the proportion of lean mass and body fat (Shah & Braverman, 2012). The syndrome called normal weight obesity has been identified among normal weight subjects whose amount of fat stores are above World Health Organization recommendations, reinforcing the role of percentage body fat (PBF) in the classification of adiposity (De Lorenzo et al., 2007). More recently, PBF has been shown to be a risk factor for total and cardiovascular mortality (Lahmann, Lissner, Gullberg, & Berglund, 2002) and cardiovascular diseases, such as coronary heart disease, independent of BMI (Dervaux, Wubuli, Megnien, Chironi, & Simon, 2008; Heitmann, Erikson, Ellsinger, Mikkelsen, & Larsson, 2000; Padwal, Leslie, Lix, & Majumdar, 2016; Romero‐Corral et al., 2010). PBF seems to be more useful in detecting early stages of cardiovascular disease risk (Yamashita et al., 2012).

The significance of the proportion and distribution of body fat to the development of cardiovascular disease and its risk factors has been evidenced in individuals without diabetes (Singh et al., 1999; Yim, Heshka, Albu, Heymsfield, & Gallagher, 2008). Obese type 2 diabetic patients have an unfavorable body fat distribution compared to obese nondiabetic patients with an increase in visceral fat (Albu et al., 2010; Gallagher et al., 2009). Excess visceral adipose tissue is known to exacerbate insulin resistance (Gallagher et al., 2009) and, consequently, increase the risk of diabetes complications. A reduction of 5% in the PBF of 171 type 2 diabetes mellitus (DM) patients was associated with improved glycemic control as assessed by HbA1c (Hancu & Radulian, 2016). Thus, a better understanding of the factors associated with PBF, especially dietary factors, is essential to help devise improved lifestyle interventions for high‐risk individuals.

Some studies have demonstrated an inverse association of PBF with carbohydrates (Miller, Lindeman, Wallace, & Niederpruem, 1990), fiber (Davis, Hodges, & Gillham, 1996; Nelson & Tucker, 1996), and protein (Soenen & Westerterp‐Plantenga, 2010), as well as some food sources, such as fruit (Davis et al., 1996). On the other hand, additional studies have reported opposite findings, especially related to dietary proteins (Koppes, Boon, Nooyens, van Mechelen, & Saris, 2009) and carbohydrates (Paul, Novotny, & Rumpler, 2004; Tucker & Kano, 1992). Dietary fat intake seems to have a strong positive influence on the increase in PBF (Miller et al., 1990; Paul et al., 2004). However, these data have not been confirmed in interventional trials to date. Additionally, many studies have evaluated the association between food sources and body fat distribution; some studies have found positive associations between food sources and abdominal obesity (e.g., increased consumption of beer, hamburgers, and French fries; Krachler et al., 2006), and others have found associations with a gynoid distribution of adipose tissue (e.g., whole grains, fruits, vegetables, vegetable oils, pasta, and low‐fat milk; Bailey, Sullivan, Kirk, & Donnelly, 2010; Krachler et al., 2006; Newby et al., 2007). Data on the association between nutrients and the body composition of type 2 diabetic patients are scarce. Regarding dietary fat intake, a recent study reported a reduction in waist circumference of type 2 diabetic patients submitted to a diet rich in omega‐3 fatty acids (Moosheer, Waldschütz, Itariu, Brath, & Stulnig, 2014). In this context, our study aimed to analyze the associations between PBF and the content and source of dietary fat in the usual diet of patients with type 2 diabetes mellitus.

## 2 METHODS

### 2.1 Patients

This cross‐sectional study recruited outpatients with type 2 diabetes who were consecutively treated at the Endocrine Division of the Hospital de Clínicas de Porto Alegre, Brazil, from June 2011 to December 2013. Our exclusion criteria were BMI >40 kg/m²; triglyceride levels >400 mg/dl; serum creatinine >2.0 mg/dl; or estimated glomerular filtration rate <30 ml/min/m³ (>Stage 3 of chronic kidney disease). Patients with other renal diseases, liver disease, and congestive heart failure or who received dietary counseling from a registered dietitian during the previous 12 months were excluded. The Strengthening the Reporting of Observational Studies in Epidemiology (STROBE) Statement was followed to perform the study (Elm et al., 2008).

### 2.2 Dietary assessment

The patients’ usual diet was assessed by means of a 3‐day weighed diet record (WDR; two nonconsecutive weekdays and 1 day of the weekend). The patients received commercial scales (1–5,000 g; Plenna) and measuring cups (25–250 ml; Pyrex), and they were given a detailed explanation of the procedures by a trained, registered dietitian. Next, patients completed a training session for a 1‐day WDR. The 3‐day WDR was recorded over 2–3 weeks and before nutritional recommendations for all patients**.** The compliance with the WDR was confirmed by a comparison of daily protein intake estimated from the 3‐day WDR (PI‐WDR) from 24‐hr urinary nitrogen output (PI‐N, collected on the third day of the WDR period; Moulin et al., 1998). According to Vaz et al. (2008), patient compliance with the WDR protocol is established if the PI‐WDR/ PI‐N ratio remains between 0.79 and 1.26.

The analysis of dietary nutrients from the 3‐day WDRs was carried out using Nutribase Clinical Nutritional Manager software (CyberSoft, USA, version 7.14, 2007). The mean values of each nutrient consumed during the 3‐day WDR were calculated. The nutritional data for frequently consumed foods were updated if necessary, and/or it was complemented with data obtained from local manufacturers of industrialized foods. The dietary variables of interest were energy, carbohydrates, protein, lipids, cholesterol, polyunsaturated fatty acids (PUFA), monounsaturated fatty acids (MUFA), and saturated fatty acids (SFA), and the following dietary fat sources were of interest: total meat (red meat, poultry, fish, and seafood), vegetable oil, and dairy products. Red meat intake was composed of beef, pork, processed meat, and viscera. Processed meat was defined as ham, salami, pate, sausage, and bologna. Additional open questions about the habit of frying food at home and reusing cooking oil were asked.

### 2.3 Anthropometric evaluation

The body weight and height of the patients (without shoes or coats) were collected with an anthropometric scale (Filizola, São Paulo, Brazil); measurements were recorded to the nearest 100 g for weight and to the nearest 0.1 cm for height. The waist circumference was measured midway between the lowest rib and the iliac crest, near the umbilicus. The hip circumference was measured at the widest point. All circumferences were measured using a nonstretch, fiberglass measuring tape (Wiso, Brazil).

The body fat composition was determined by 8‐point bioelectrical impedance (InBody 230, Biospace Co. Ltd., Seoul, Korea). The body was analyzed in five segments: arms (right and left), legs (right and left), and trunk. The total percentage of body fat determined by bioelectrical impedance was used in the analysis. The test was performed after 8 hr of fasting. The patients had to have emptied their bladders at a maximum of 30 min before the exam, not have consumed alcoholic or caffeinated beverages for 48 hr, and not have exercised for 24 hr. The patients were asked to take their day's medications after the test (Clinical Research Team in Biospace Co., Ltd, 2016).

### 2.4 Clinical and laboratory evaluation

Blood pressure was measured twice using a digital sphygmomanometer (OMRON^®^ Automatic Blood Pressure Monitor, Model HEM‐705CP, Vernon Hills, Illinois 60061), and the average of the two readings was used. Hypertension was defined as blood pressure ≥140/90 mmHg or by the use of antihypertensive medication on at least two separate occasions. Physical activity level was evaluated using the International Physical Activity Questionnaire (IPAQ) (2014^2014^). Renal function was evaluated by serum creatinine and urinary albumin excretion (Gross et al., 2005). The glomerular filtration rate (GFR) was estimated by the CKD‐EPI (*Chronic Kidney Disease Epidemiology Collaboration*) equation (Levey et al., 2009). Diabetic kidney disease was defined as the presence of 24‐hr urinary albumin excretion higher than 30 mg (always confirmed in a second measurement) and/or a GFR lower than 60 ml/min/m² (Levey et al., 2011). Socioeconomic status was evaluated according to the Criteria of Economic Classification Brazil (The Criteria of Economic Classification Brazil, 2008).

A fasting blood sample was drawn on the same day as the anthropometric evaluation. Glycemic control was assessed through serum glucose (glucose‐peroxidase colorimetric enzymatic method, Biodiagnostica^®^) and glycated hemoglobin (high precision chromatography, Merck‐Hitachi 9100 apparatus). The lipid profile consisted of total cholesterol and triglycerides using a colorimetric assay. HDL cholesterol was measured using a direct enzymatic method. LDL cholesterol was calculated using the Friedewald formula. Serum creatinine was measured by the Jaffe method; urea, by kinetic UV assay. Urinary albumin excretion was evaluated by immunoturbidimetry (Kit MICROALB, AMES, USA; Zelmanovitz, Oliveira, Lhullier, Gross, & Azevedo, 1994).

### 2.5 Statistical analyses

Student's *t* test, Mann–Whitney's *U* test, and the exact Fisher or chi‐square tests were utilized. A multivariate analysis was performed by Poisson's regression to estimate the dietary factors associated with increased PBF (dependent variable). Different models were generated to evaluate the association of each dietary fat component and food source (independent variables) with increased PBF. The independent variables were selected based on their significance (*p* < 0.10) via a univariate analysis or according to their biological relevance. The dietary intake of each analyzed nutrient or food source was also divided into tertiles to assess possible differences in PBF across their intake. The data were expressed as the mean ± standard deviation or as median (P25‐P75) unless otherwise stated. The level of significance adopted was 5%. SPSS 18.0 (SPSS^®^, Chicago, IL, USA) was used for the analyses.

### 2.6 Ethics, consent, and permissions

This study was conducted in accordance with the guidelines laid out in the Declaration of Helsinki, and all procedures involving patients were approved by the Hospital Ethics Committee. Written informed consent was obtained from all patients.

## 3 RESULTS

### 3.1 Patients

Two hundred and twenty‐seven patients were recruited, but 39 were excluded because of the following reasons: eight refused to participate, two had difficulty traveling to and from the hospital, 20 patients were not capable of performing the WDR, one was participating in another study, and eight received a diagnosis of other active disease. A total of 188 type 2 diabetic patients (107 women and 81 men) entered the protocol and underwent clinical, anthropometric, nutritional, and laboratory evaluations. Their mean age was 62.5 ± 8.8 years, and 76.7% of the patients reported themselves as white. The mean time of a known diabetes diagnosis was 14.2 ± 9.6 years; 81.8% had hypertension, and 43.9% had diabetic kidney disease. Regarding antihyperglycemic treatment, 1.6% did not use any medication, 50% were on insulin, 83.1% used metformin, and 41.8% used other antidiabetic medications. Forty‐six percent of patients were classified as sedentary. With regard to socioeconomic status, 41% were classified as in the lower stratum. The mean BMI was 29.3 ± 3.8 kg/m² (11.2% were eutrophic; 48.4%, overweight; and 40.4%, obese), and the mean waist circumference was 100.7 ± 9.4 cm in women and 101.5 ± 7.8 cm in men. Women had a higher mean PBF (39.8 ± 5.9%) than men (26.6 ± 7.1%; *p* = 0.001).

The patients were divided according to the mean PBF for each gender: high PBF group (PBF >39.5% for women; PBF >26.6% for men) and low PBF group. As expected, BMI was higher in the group with high PBF than that in the group with low PBF, as shown in Table 1. There was no difference in the other demographic and laboratory characteristics between the groups.

**Table 1:** Clinical and laboratory characteristics of type 2 diabetic patients divided according to the mean PBF

	Percentage body fat		*p*
	Low	High	
*N*	85	103	
Age (years)	62.0 ± 9.3	62.9 ± 8.4	0.501
Gender (male; %)	37 (43.0)	44 (42.7)	0.999
Known duration of diabetes (years)	14.5 ± 9.7	13.9 ± 9.6	0.716
Race (White)	62 (72.1)	83 (80.6)	0.226
Hypertension (%)	61 (71.8)	87 (84.5)	0.120
Diabetic kidney disease (%)	37 (43.5)	45 (43.7)	0.999
Sedentary (%)	34 (38.6)	37 (35.9)	0.747
Insulin therapy (%)	35 (41.2)	40 (38.8)	0.624
Use of metformin (%)	61 (71.8)	63 (61.2)	0.503
BMI (kg/m²)	27.3 ± 2.9	31.0 ± 3.5	<0.001
Waist circumference (cm)			
Men	97.8 ± 8.1	104.9 ± 5.7	<0.001
Women	96.2 ± 7.8	104.4 ± 9.0	<0.001
Waist‐to‐hip ratio			
Men	0.98 ± 0.06	1.03 ± 0.05	<0.001
Women	0.95 ± 0.07	0.94 ± 0.06	0.772
Glycemia (mg/dl)	160 ± 60	156 ± 62	0.655
Glycated hemoglobin (%)	8.3 ± 2.0	8.1 ± 1.6	0.458
Total cholesterol (mg/dl)	172 ± 34	178 ± 43	0.322
HDL cholesterol (mg/dl)	44 ± 11	44 ± 8	0.950
LDL cholesterol (mg/dl)	98 ± 30	99 ± 34	0.893
Triglyceride (mg/dl)	130 (89–197)	145 (96–191)	0.307
UAE (mg/24 hr)	9 (3–34.3)	14 (3–48.1)	0.294
Creatinine (mg/dl)	0.91 ± 0.26	0.88 ± 0.23	0.382

*Notes*

Data are presented as mean ± *SD*, median (interquartile range), or number of cases to total of patients ratio in each group (%).

BMI: body mass index; PBF: percentage body fat; HDL: high density lipoprotein; LDL: Low density lipoprotein; UAE: urinary albumin excretion.

### 3.2 Associations between dietary fat composition and food source and percentage body fat

The dietary intake characteristics of the groups with higher and low PBF are presented in Table 2. There was no difference between the groups regarding energy and nutrient intake. In the high PBF group, a greater proportion of red meat intake in relation to total meat (52.0% vs. 42.3%; *p* = 0.036), especially processed meat (5.4% vs. 3.0%; *p* = 0.010), was observed when compared to the low PBF group. The high PBF group consumed more saturated fat from red meat (2.1% of energy) than the low PBF group (1.5% of energy; *p* = 0.032). The reuse of frying oil was more frequent in the group with high PBF (31.2 vs. 11%; *p* = 0.011) when compared to the low PBF group.

**Table 2:** Dietary characteristics of type 2 diabetes patients divided according to low and high PBF

	Percentage body fat		*p*
	Low	High	
*N*	85	103	
Energy (Kcal)	1,883 ± 473	1,897 ± 579	0.855
Proteins (% of energy)	18.5 ± 3.6	18.4 ± 3.9	0.899
Carbohydrates (% of energy)	48.1 ± 7.8	47.5 ± 8.1	0.616
Fat (% of energy)	33.9 ± 7.8	34.1 ± 7.7	0.846
Cholesterol (mg/day)	192 (139–240)	218 (136–273)	0.152
Trans FA (% of energy)	0.84 (0.55–1.16)	0.89 (0.60–1.24)	0.474
SFA (% of energy)	9.24 ± 2.53	8.94 ± 2.46	0.408
MUFA (% of energy)	11.75 ± 3.24	11.72 ± 3.09	0.941
PUFA (% of energy)	10.26 ± 4.28	10.53 ± 3.82	0.658
Fiber (g/1,000 Kcal)	11.8 ± 4.8	11.7 ± 4.4	0.816
Vegetable oil (ml/day)	22.9 (15.0–33.5)	22.5 (15.0–32.2)	0.609
Dairy products (g/1,000 Kcal)	152 (67.5–212.8)	109 (46.8–180.1)	0.059
SFA from dairy (g/1,000 Kcal)	2.03 (0.68–3.0)	1.6 (0.48–3.11)	0.568
Red meat (g/1,000 Kcal)	43.4 (21.7–64.4)	55.5 (27.2–77.9)	0.056
Red meat (% of total meat)	42.3 (20.2–68.4)	52.0 (31.3–100.0)	0.036
Processed meat (g/1,000 Kcal)	3.0 (0.0–10.0)	7.5 (0.0–15.9)	0.019
Processed meat (% of total meat)	3.0 (0.0–8.1)	5.4 (0.0–13.1)	0.010
SFA from red meat (g/1,000 Kcal)	1.7 (0.5–3.2)	2.3 (1.0–4.5)	0.032
Fry food at home (yes) – *n* (%)	56 (65.9%)	70 (68.0%)	0.467
Reuse of frying oil (yes) – *n* (%)	11 (14.1%)	29 (31.2%)	0.011

*Notes*

Data are presented as mean ± *SD*, median (interquartile range), or number of cases to total of patients ratio in each group (%).

MUFA: monounsaturated fatty acid; PBF: percentage body fat; PUFA: polyunsaturated fatty acid; SFA: saturated fatty acid.

In the Poisson analysis, the intake of red meat (% of total meat) was positively associated with the presence of high PBF (PR = 1.008 [95% CI = 1.002–1.013]; *p* = 0.006) after adjustment for age, socioeconomic status, gender, insulin therapy, metformin use, sedentary habits, diabetes kidney disease, energy intake, and WDR compliance. When processed meat intake (% of total meat) was separately analyzed, it was also positively associated with the presence of high PBF (PR = 1.010 [95% CI = 0.998–1.022]; *p* = 0.098), but it did not reach statistical significance after adjustment for the same covariates. In another model, the reuse of frying oil was associated with high PBF (PR = 1.670 [95% CI = 1.240–2.249]; *p* = 0.001), and it was adjusted for the same variables.

### 3.3 Analyses according to the tertiles of daily fat intake and its sources

**Figure 1:**
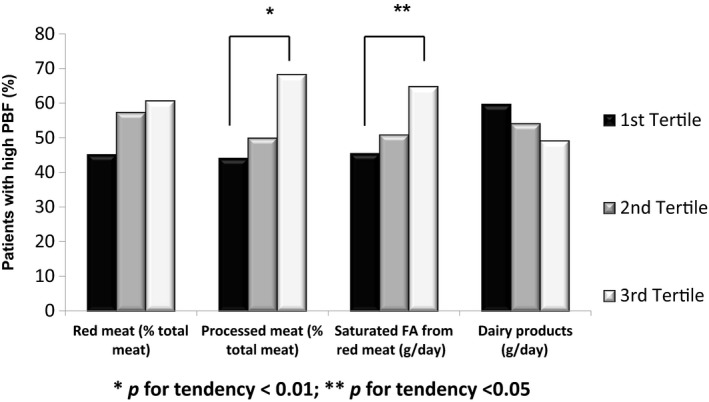
Proportion of type 2 diabetes patients with higher percentage body fat (PBF) according to the tertiles of intake. * *p* < 0.01; ** *p* = 0.05

The nutrients and food groups were divided into tertiles of intake. The tertiles of processed meat (% of total meat) were linearly associated with an increased proportion of patients with high PBF as follows: upper tertile: 68.3%, second tertile: 50.0%, and lower tertile: 44.1% of patients with high PBF (*p* for trend = 0.007), as shown in Figure 1. The same was true for SFA from red meat: upper tertile: 64.8%, second tertile: 50.9%, and lower tertile: 45.5% of patients with high PBF (*p* for trend = 0.044; Figure 1). For red meat intake (% of total meat), the proportion of patients with high PBF was 60.7%, 57.4%, and 45.2% in the upper, second, and lower tertile, respectively, but these values were not significantly different (*p* = 0.085). An inverse linear association was observed with dairy product consumption. In the upper tertile, 59.7% of patients presented high PBF, 54.1% in the second tertile, and 49.1% in the lower tertile (*p* for trend = 0.244).

In the Poisson analysis, the upper tertile of red meat intake was positively associated with the presence of high PBF (dependent variable; PR = 1.294 [95% CI = 1.059–1.582]; *p* = 0.012) after adjustment for age, socioeconomic status, gender, insulin therapy, metformin use, sedentary habits, diabetes kidney disease, energy intake and WDR compliance. The intake of processed meat in the upper tertile was also associated with high PBF (PR = 1.522 [95% CI = 1.226–1.891]; *p* = 0.001) after adjusting for the same covariates.

## 4 DISCUSSION

The present study reported a positive association between dietary sources of saturated fat, such as red meat and processed meat, and PBF of patients with type 2 diabetes, independent of other biological factors. The patients with an increased PBF consumed more saturated fat from red meat than those with a low PBF, although this association was not confirmed in the multivariate analysis. Moreover, our study found a strong association between the habit of reusing frying oil and PBF.

In many studies with nondiabetic subjects, total meat intake was associated with markers of adiposity, such as BMI and waist circumference, but not with PBF (Kahn et al., 1997; Wang & Beydoun, 2009; Williams, 2012). Williams et al. analyzed the dietary pattern of men and women with different levels of physical activity and observed that a dietary pattern with higher meat and lower fruit intake was significantly and consistently associated with a greater BMI and waist circumference at all physical activity levels (Williams, 2012). In addition, data from the National Health and Nutrition Examination Survey (NHANES) suggest a consistent positive association between meat consumption and obesity and central obesity (Wang & Beydoun, 2009). Additionally, regarding the intake of specific subtypes of meat, a previous study with white, non‐Hispanic, healthy adults reported that a 10‐year change in BMI was positively associated with red meat consumption (Kahn et al., 1997). Furthermore, in a study with two large population cohorts from the Nurses’ Health Study and from the Health Professionals Follow‐up Study, it was observed that an increase in red meat consumption over time was associated with an elevated risk for type 2 diabetes, although this association was partly mediated by an increase in body weight (Pan et al., 2013). In the baseline analysis of a cohort study with 3,902 older adults from both genders, those with the highest meat intake also had the highest BMI. However, after 14 years of follow‐up, red meat intake was not associated with weight change (Gilsing et al., 2012).

Although large longitudinal studies have presented a positive relationship between red meat consumption and adiposity, this association was not confirmed in one recent randomized controlled trial (Murphy et al., 2014). Murphy and colleagues evaluated the effect of three types of meat (red meat: lean pork and beef; white meat: chicken) on the adiposity of normal subjects who were followed for 9 months. The authors did not observe differences in any index of adiposity, and there was no change in lean mass among the groups over time (Murphy et al., 2014). Nevertheless, there is a lack of large interventional studies analyzing the effect of meat consumption on adiposity.

In the present study, processed meat intake was related to high PBF, although a low quantity of processed meat was consumed by our sample of patients. Similar findings were observed among European men and women. A dietary pattern characterized by a low consumption of processed meat, white bread, margarine, and soft drinks and a high consumption of fruits and dairy products was associated with a lower gain in waist circumference and BMI (Romaguera et al., 2011). This subtype of meat is mainly a source of SFA (Hansen et al., 2012) and an important source of sodium (Micha, Wallace, & Mozaffarian, 2010).

Red and processed meats have higher proportions of SFA when compared to other types of meat, such as poultry and fish (white meat), and this is the main difference among these types of meat. For this reason, the SFA content of red meat could possibly be the main factor associated with an increased PBF. In our study, patients with an increased PBF consumed more saturated fat from red meat than those with a low PBF, despite the lack of statistical significance in the multivariate analysis. In a 4‐week randomized crossover trial, the substitution of an SFA‐rich diet with a MUFA‐rich diet resulted in a small but significant reduction in fat mass in overweight and obese men (Piers, Walker, Stoney, Soares, & O'Dea, 2003). It is suggested that SFA are probably more obesogenic than MUFA and PUFA are (Krishnan & Cooper, 2014). Unsaturated fat appears to be more metabolically beneficial than SFA, as evidenced by a high‐diet‐induced thermogenesis and fat oxidation following high‐fat meals or diets (Krishnan & Cooper, 2014).

The consumption of other sources of SFA, such as dairy products and vegetable oils, was analyzed in our population. The patients with low PBF showed a tendency to consume more dairy products than those with increased PBF. A longitudinal evaluation of the Framingham cohort found an inverse association between dairy consumption and adiposity, evaluated by weight, BMI, and waist circumference (Wang et al., 2014). Additionally, our study did not observe a difference in total SFA intake between the higher and low PBF groups. One possible explanation for these findings is the difference in carbon chain length between fractions of SFA in dairy products and in red meat. Moreover, the reuse of frying oil was strongly associated with a high PBF in our sample of type 2 diabetic patients. The deep‐frying process leads to oxidation, polymerization, and hydrolysis of vegetable oils (Choe & Min, 2007), and these reactions increase with frying frequency (Koh & Surh, 2015). As a result, free fatty acids and toxic compounds, such as benzene, are produced (Choe & Min, 2007) and could increase health problems. Soybean oil was the most common fat used for cooking in our sample. When heated in the deep‐frying process, this vegetable oil increases in the proportion of saturated fatty acids (Lopes, Aued‐Pimentel, Caruso, Jorge, & Ruvieri, 2004). Our work is the first study describing the association between the reuse of frying oil and PBF; therefore, more longitudinal studies are needed to support these findings.

In the present study, no association was observed between high PBF and unsaturated FA, such as MUFA and/or PUFA, nor trans fatty acids. In fact, regarding polyunsaturated fat, studies are controversial. A cohort study with 1,212 twins from Denmark showed that the intake of n‐3 PUFA, in particular ɑ‐linolenic acid, was beneficially associated with body fat (Lund et al., 2013). Moreover, Ramel and colleagues observed a dose–response relationship between cod consumption as a source of n‐3 PUFA and weight loss (Jakobsen et al., 2012). Nevertheless, other authors did not find an additional beneficial effect of these nutrients or their dietary sources on total body or abdominal fat (Ramel, Jonsdottir, & Thorsdottir, 2009; Tapsell et al., 2013). A randomized parallel study with 118 obese Australian adults tested three interventions: low calorie dietary advice + placebo, low calorie dietary advice emphasizing fish meat + placebo, or low calorie dietary advice emphasizing fish meat + long chain n‐3 PUFA supplements. All dietary interventions reduced weight and body fat similarly (Ramel et al., 2009). In the Nurses’ Health Study and Health Professionals’ Follow‐up Study, an increase of 1% and 2% of energy intake from total trans fatty acids was associated with a weight and waist circumference gain, respectively (Field, Willett, Lissner, & Colditz, 2007; Koh‐Banerjee et al., 2003). On the other hand, trans fatty acid intake, specifically from ruminant dairy and meat products, was inversely associated with body weight (Hansen et al., 2012). However, in the present study, we did not find any association between trans fatty acid intake and PBF, perhaps because of the relatively low consumption of this fatty acid in our population compared with previous studies (Field et al., 2007; Koh‐Banerjee et al., 2003).

The present study demonstrated the association between red meat intake and the reuse of frying oil with PBF, specifically, in type 2 diabetic patients. As far as we know, data about the relationship between the food source of dietary fat and adiposity are scarce in diabetic people. One study evaluated the association of some nutrients and PBF in type 2 diabetic patients and demonstrated that specific proportions of carbohydrates and fats affect the topography of fat distribution in these patients (Paniagua et al., 2007).

One potential limitation of our study was the use of bioimpedance for the PBF evaluation instead of a more accurate technique such as dual‐energy X‐ray absorptiometry. However, bioimpedance with an 8‐point tactile electrode system is a well‐validated technique in several populations (Karelis, Chamberland, Aubertin‐Leheudre, & Duval, 2013), including our type 2 DM population (Bello, Pavinatto, Duarte, Graciano, Azevedo, & Zelmanovitz, 2015), and we excluded individuals with BMI >40 kg/m² since bioimpedance is not as precise with extreme BMIs (Bedogni et al., 2013). Another limitation was the relatively small sample size of our study; a larger sample would give more statistical power to confirm other associations. A third limitation of our study was the large proportion of people with diabetic kidney disease. Since this clinical condition may influence body composition (Hoffmann, Senior, Jackson, Jindal, & Mager, 2016), we decided to adjust the Poisson regression models for the presence of diabetic kidney disease, but the results were not changed. Additionally, the measurement of a biological marker of fat intake would perhaps have reinforced our results. Serum fatty acids have been used to evaluate fat intake in patients with type 2 diabetes (Perassolo et al., 2008). We could not collect these markers. Nonetheless, our dietary data were accurately analyzed. We used a standardized 3‐day weighed diet record, which included a 24‐hr urinary urea measurement to confirm the dietary intake estimated from the records (Moulin et al., 1998; Vaz et al., 2008). This tool has been largely used to confirm dietary compliance in studies with diabetic patients (Almeida et al., 2008; Gross et al., 2002).

In conclusion, this cross‐sectional study reported a positive association between dietary sources of SFA**,** especially red and processed meat, and PBF in type 2 diabetic patients. Further studies, prospective and interventional, are needed to strengthen these findings.

## ACKNOWLEDGMENT

This paper received support from Pró‐Reitoria de Pesquisa da UFMG.

## CONFLICT OD INTEREST

The authors declare that they have no conflict of interests.

## TRANSPARENCY DECLARATION

The lead author affirms that this manuscript is an honest, accurate, and transparent account of the study being reported. The reporting of this work is compliant with STROBE guidelines. The lead author affirms that no important aspects of the study have been omitted and that any discrepancies from the study as planned (HCPA GPPG Number 11‐0250) have been explained.

## APPENDIX 1 STROBE statement—Checklist of items that should be included in reports of *cross‐sectional studies*

### 1.1

**Table:**

	Item No	Recommendation
Title and abstract	1	Indicate the study's design with a commonly used term in the title or the abstract Provide in the abstract an informative and balanced summary of what was done and what was found
Introduction		
Background/rationale	2	Explain the scientific background and rationale for the investigation being reported
Objectives	3	State specific objectives, including any prespecified hypotheses
Methods		
Study design	4	Present key elements of study design early in the paper
Setting	5	Describe the setting, locations, and relevant dates, including periods of recruitment, exposure, follow‐up, and data collection
Participants	6	Give the eligibility criteria, and the sources and methods of selection of participants
Variables	7	Clearly define all outcomes, exposures, predictors, potential confounders, and effect modifiers. Give diagnostic criteria, if applicable
Data sources/measurement	8^a^	For each variable of interest, give sources of data and details of methods of assessment (measurement). Describe comparability of assessment methods if there is more than one group
Bias	9	Describe any efforts to address potential sources of bias
Study size	10	Explain how the study size was arrived at
Quantitative variables	11	Explain how quantitative variables were handled in the analyses. If applicable, describe which groupings were chosen and why
Statistical methods	12	Describe all statistical methods, including those used to control for confounding Describe any methods used to examine subgroups and interactions Explain how missing data were addressed If applicable, describe analytical methods taking account of sampling strategy Describe any sensitivity analyses
Results		
Participants	13^a^	Report numbers of individuals at each stage of study—for example numbers potentially eligible, examined for eligibility, confirmed eligible, included in the study, completing follow‐up, and analysed Give reasons for non‐participation at each stage Consider use of a flow diagram
Descriptive data	14^a^	Give characteristics of study participants (e.g., demographic, clinical, social) and information on exposures and potential confounders Indicate number of participants with missing data for each variable of interest
Outcome data	15^a^	Report numbers of outcome events or summary measures
Main results	16	Give unadjusted estimates and, if applicable, confounder‐adjusted estimates and their precision (e.g., 95% confidence interval). Make clear which confounders were adjusted for and why they were included Report category boundaries when continuous variables were categorized If relevant, consider translating estimates of relative risk into absolute risk for a meaningful time period
Other analyses	17	Report other analyses done—for example analyses of subgroups and interactions, and sensitivity analyses
Discussion		
Key results	18	Summarise key results with reference to study objectives
Limitations	19	Discuss limitations of the study, taking into account sources of potential bias or imprecision. Discuss both direction and magnitude of any potential bias
Interpretation	20	Give a cautious overall interpretation of results considering objectives, limitations, multiplicity of analyses, results from similar studies, and other relevant evidence
Generalisability	21	Discuss the generalisability (external validity) of the study results
Other information		
Funding	22	Give the source of funding and the role of the funders for the present study and, if applicable, for the original study on which the present article is based

*Note*

Give information separately for exposed and unexposed groups.
